# Mechanistic Basis for the Role of Phytochemicals in Inflammation-Associated Chronic Diseases

**DOI:** 10.3390/molecules27030781

**Published:** 2022-01-25

**Authors:** Brianna Cote, Fawzy Elbarbry, Fiona Bui, Joe W. Su, Karen Seo, Arthur Nguyen, Max Lee, Deepa A. Rao

**Affiliations:** 1 College of Pharmacy, Oregon State University, Portland, OR 97201, USA; coteb@oregonstate.edu; 2 School of Pharmacy, Pacific University, Hillsboro, OR 97123, USA; Fawzy.elbarbry@pacificu.edu (F.E.); fionabui34@gmail.com (F.B.); Karen.park.seo@pacificu.edu (K.S.); nguy8256@g.pacificu.edu (A.N.); lee7829@g.pacificu.edu (M.L.); 3 School of Pharmacy, West Coast University, Los Angeles, CA 90004, USA; JSu@westcoastuniversity.edu

**Keywords:** natural products, complementary and alternative therapies, inflammation associated diseases, natural product drug interactions

## Abstract

Chronic inflammatory diseases occur in a large portion of the population and are associated with a poor diet. Key natural products found in fruits and vegetables may assist in lowering inflammation associated with chronic diseases such as obesity, diabetes, cardiovascular diseases, and cancer. This review seeks to examine the roles of several natural products, resveratrol (RES), quercetin (QUE), curcumin (CUR), piperine (PIP), epigallocatechin gallate (EGCG), and gingerol (GIN), in their ability to attenuate inflammatory markers in specific diseases states. Additionally, we will discuss findings in past and ongoing clinical trials, detail possible phytochemical–drug interactions, and provide a brief resource for researchers and healthcare professionals on natural product and supplement regulation as well as names of databases with information on efficacy, indications, and natural product–drug interactions. As diet and over-the-counter supplement use are modifiable factors and patients are interested in using complementary and alternative therapies, understanding the mechanisms by which natural products have demonstrated efficacy and the types of drugs they interact with and knowing where to find information on herbs and supplements is important for practicing healthcare providers and researchers interested in this field.

## 1. Introduction

Chronic inflammation is long-term, low-grade, asymptomatic inflammation that can occur due to various conditions such as chronic infections, low physical inactivity, obesity, poor diet, disturbed sleep patterns, isolation, and stress [1]. If unaddressed and chronic inflammation persists, it can lead to diabetes, cancer, depression, metabolic syndrome, neurodegenerative diseases, and autoimmune diseases [1]. Sixty percent of all deaths around the world occur from conditions associated with chronic inflammatory diseases, such as obesity, diabetes, cardiovascular disorders, and cancer [2]. The impact of diet on inflammation-associated disease is well-studied [3], and poor diet is associated with chronic degenerative diseases [4]. Per the World Health Organization (WHO), the consumption of fruits and vegetables and lower levels of red meat could reduce cardiovascular diseases and cancer risk [5]. Numerous studies depict a healthy diet as a modifiable determinant of chronic diseases, resulting in the attenuation of oxidative stress [6], a decrease in cancer-specific mortality [7], and suppression of chronic inflammation [8].

Inflammation is a defense mechanism that is intended to eliminate infectious agents and restore affected tissues to homeostasis [9]. Inflammation at the tissue level typically presents as swelling, heat, pain, redness, and potential loss of tissue function [10]. Inflammation can be acute or chronic, and the inflammatory process follows a similar mechanism in both phases. When a trigger is detected, cell surface receptors will lead to the activation of the inflammatory cascade, leading to the release of inflammatory markers and the recruitment of inflammatory cells [10]. In the case of acute inflammation, the process terminates on the removal or resolution of the trigger; in the case of chronic inflammation, the body is unable to repair and/or overcome the trigger.

An acute trigger results in the initiation of innate immunity, resulting in inflammation in minutes, clearing infections in hours to days [11]. The innate immune system consists of hematopoietic origin cells, which respond to early-stage infections [11]. Foreign microbe detection triggers an inflammatory process and receptor-mediated cytokine production, resulting in vasodilation [12]. Acute inflammation often results in neutrophil migration and increased blood flow and permeability [12]. Chemokines cause rapid neutrophil infiltration and macrophage recruitment [12]. These cells initiate phagocytosis and degrade foreign microorganisms, resulting in infection clearance [12].

On the other hand, chronic inflammation underlies a variety of disease states, including, but not limited to, cancer, heart disease, diabetes, chronic kidney disease, neurodegenerative disorders, and autoimmune conditions [1]. Chronic inflammation may manifest due to persistent microbial infection, recurring tissue injury, or failure of acute inflammation to subside and may persist for years [13]. Inflammasomes are a group of cytosolic multiprotein complexes responsible for initiating and propagating the inflammatory process [14]. Inflammasome complexes are composed of the nucleotide-binding oligomerization domain (NOD), leucine-rich repeating receptors (NLRs) containing pyrin domain 3 (NLRP3), the caspase recruitment domain (ASC), and caspase-1 (Casp1) [15]. Inflammasome activation protects against pathogenic invasion. Immune cell receptors recognize microbes via pathogen-associated molecular patterns (PAMPs) or danger-associated molecular patterns (DAMPs) using pattern recognition receptors (PRRs) such as toll-like receptors (TLRs), which stimulate inflammatory cytokine and chemokine expression [15]. Exogenous triggers such as persistent microbial infection and endogenous triggers such as poor diet, obesity, stress, disturbed sleep patterns, recurring tissue injury, and failure of acute inflammation to subside activate PAMPs and DAMPs, respectively. Inflammasome receptors detect PAMPs and DAMPs, and simultaneous ligation of receptors activates the NLRP3 inflammasome [16]. Activation requires a priming signal to detect stimuli and release pro-inflammatory cytokines and an activation signal to cleave cytokines into their mature form, activating the inflammasome [17]. 

**Figure 1:**
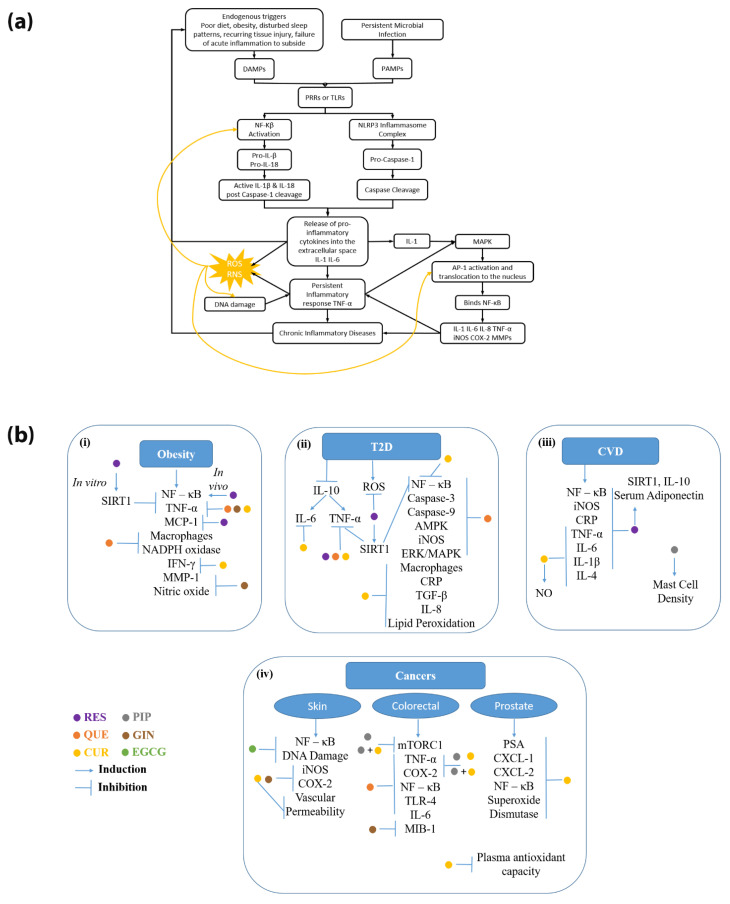
(**a**) Depiction of chronic inflammation pathways and their intersection with chronic inflammatory diseases. (**b**) Common inflammatory markers associated with various chronic inflammatory diseases and the evidence of where phytochemicals can exert mitigating effects in these disease states.

The nuclear factor kappa-light-chain-enhancer of activated B-cells (NF-κB) pathway is essential for the expression of pro-inflammatory genes in the pathogenesis of chronic inflammatory diseases [18]. The NLRP3 inflammasome activates Casp1, cleaving pro-interleukin (IL)-1β and pro-IL-18 into their active forms, IL-1β and IL-18 [17]. Persistent inflammatory cell infiltration may cause leukocyte-induced injury, triggering loss of function or tissue damage [19]. Inflammation progression produces reactive oxygen species (ROS) and reactive nitrogen species (RNS), which damage DNA [20,21]. ROS and inefficient DNA repair can cause a positive feedback loop, resulting in further DNA damage, ROS production, and genomic instability [22]. Further, oxidative stress can cause the overexpression of NF-kB and activator protein 1 (AP-1), escalating inflammation [23,24]. Chronic activation of the inflammatory response can stimulate transforming growth factor β-1 (TGF-β1), inducible nitric oxide synthase (iNOS), and cyclooxygenase-2 (COX-2), which play central roles in inflammatory disease development [23,25]. Matrix metalloproteinases (MMPs), a family of endopeptidases, also play a role in inflammation [26]. MMPs are active in normal and disease-related inflammation but have increased expression in inflammatory diseases and assist in mediating inflammatory signals from cytokines and chemokines [26]. Comprehensive reviews describe roles of these cytokines in inflammation in chronic diseases, including cancer [27], cardiovascular diseases [28], and obesity [29]. Thus, these diseases are strongly associated with the NLRP3 inflammasome, creating positive feedback loops and amplifying levels of inflammation and organ damage. Figure 1a provides an overview of how chronic inflammation can lead to the various disease states, which, in turn, can lead to further inflammatory events.

**Figure 2:**
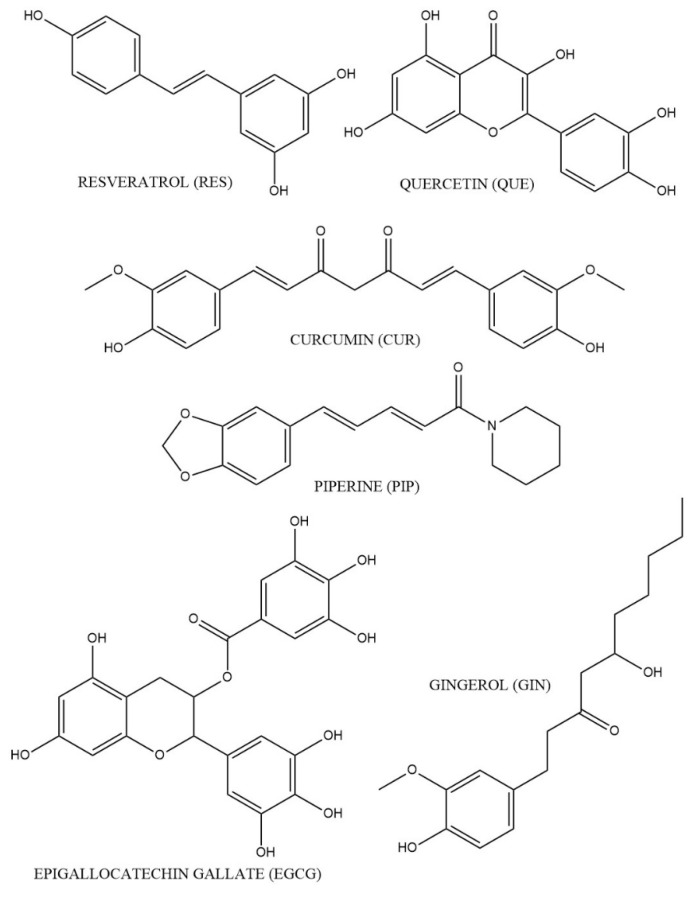
Chemical structures of selected phytochemicals.

Natural systems of medicine like Ayurveda and traditional Chinese medicine utilize extracts or teas of natural products to treat or cure various disease states [30]. Phytochemicals found in plants can defend against diseases and inflammation by acting as antioxidants. However, the use of phytochemicals for the treatment of chronic inflammation remains underutilized in western medicine. Research has been conducted on several phytochemicals found in many foods, including resveratrol (RES), quercetin (QUE), curcumin (CUR), piperine (PIP), epigallocatechin gallate (EGCG), and gingerol (GIN), for their medicinal effects (Figure 2). Phytochemicals such as RES and QUE are found in grapes [31]. CUR is a component of the turmeric root [32]. PIP is a phytochemical found in black peppers [33]. EGCG is a catechin in green tea [34]. GIN is an active constituent of the ginger root [35]. These products exhibit anti-inflammatory properties and may alleviate inflammation associated with chronic diseases. In this review, we aim to elucidate the relationship between the effects of phytochemicals on inflammation in major inflammation-induced chronic diseases, discuss potential drug–phytochemical interactions, and provide a brief overview on natural product regulations and resources for interested researchers and healthcare providers.

## 2. Current Research Supporting Phytochemical Use in Various Chronic Diseases

In the sections below, selected disease states are discussed in terms of how chronic inflammation leads to and continues to perpetuate the disease state. Evidence for how phytochemicals may ameliorate these diseases by modulating inflammatory responses seen in chronic inflammation is presented, and a summary version is depicted in Figure 1b to compare the various molecular markers and triggers related to chronic inflammation (Figure 1a).

### 2.1. Diseases Related to Metabolic Syndrome 

According to the National Heart, Lung, and Blood Institute at the NIH, metabolic syndrome is defined as a group of risk factors that lead to biochemical and physiological abnormalities, resulting in the development of type II diabetes, cardiovascular disease, and an increased risk for stroke [36]. There are five metabolic risk factors that may pre-dispose an individual to metabolic syndrome. These risk factors include a large waistline, high triglyceride level, low high-density lipoprotein level, high blood pressure, and high fasting blood sugar [36]. The presence of 3 or more of these metabolic risk factors results in a diagnosis of metabolic syndrome. Thus, it is important to understand the effect and role that phytochemicals may play in ameliorating these risks. 

In a recent clinical trial, older adults (13 males and 9 females) diagnosed with metabolic syndrome and on concurrent therapeutics to treat their conditions were provided with supplements containing RES + PIP + α-tocopherol (FRAMINTROL^®^) [37]. Over the course of three months, the subjects were evaluated for the inflammatory state by tracking C-reactive protein (CRP), an inflammatory marker, ferritin in plasma, and oxygen consumption and chemiluminescence in neutrophils. At the end of the study, significantly lower ferritin, CRP, and oxygen consumption from baseline were noted in these subjects [37]. Neutrophil chemiluminescence is an indicator of oxidative stress in cells as neutrophils are the main intracellular source of the superoxide anion and hydrogen peroxide associated with the inflammatory process and seen in metabolic syndrome. A significant decrease in chemiluminescence may be indicative of anti-inflammatory effects post-treatment [37]. While the results of this study are promising, the small sample size and the concurrent use of therapeutics make the interpretation of the findings unclear.

#### 2.1.1. Obesity

Obesity prevalence has increased significantly since the 1980s, almost tripling since 1975 [38]. According to the WHO, in 2016, 1.9 billion adults were overweight, and of these, 650 million suffered from obesity [38]. Weight gain results in adipogenesis, a mechanism by which preadipocytes differentiate into mature adipocytes. These elevated adipocytes generate free fatty acids, adipokines, and chemical messengers that affect appetite, lipid metabolism, and inflammation [39]. Differentiation of preadipocytes depends on morphological changes, accumulation of lipids, cell cycle arrest, and adipokine expression [39]. Adipose tissue macrophages become polarized and become pro-inflammatory macrophages, which, in turn, leads to the upregulation of proinflammatory cytokine production and release, ultimately impairing preadipocyte differentiation and increasing lipid storage [39,40]. Thus, obesity is complemented by inflammation, which promotes proinflammatory cytokine expression, including tumor necrosis factor-α (TNF-α), IL-6, and IL-1β, which may be linked to adipose tissue expansion [39]. 

Evidence shows that RES can mediate obesity by inhibiting the activity of NF-κB and suppressing TNF-α through nicotinamide adenine dinucleotide (NAD)-dependent protein deacetylase sirtuin-1 (SIRT1) activation [41,42]. RES also attenuates TNFα-induced monocyte chemoattractant protein (MCP)-1 expression in primary human adipocytes [43]. Although the in vitro data looks promising, a double-blind, randomized trial in a small population of obese men suggests that while RES supplementation significantly decreases adipocyte size, RES supplementation results in an increased inflammatory response with upregulation in T-cell signaling and NF-κB [44]. Supplementation also did not show beneficial metabolic effects in normal glucose-tolerant non-obese women [45]. Thus, the effects of RES can be both pro- and anti-inflammatory and may be due to the effects of RES on various molecular pathways [46].

QUE inhibits lipid accumulation during adipogenesis by suppressing adipogenic and lipogenic transcription factors and inhibiting adipokine secretion in vitro and in vivo [47]. QUE inhibits inflammation by activating mTOR and p7056K and inhibiting the MAPK signaling pathway, decreasing body weight in zebrafish and mice [47]. QUE also stimulates anti-inflammatory cytokine IL-10, inhibiting pro-inflammatory cytokines [47]. In high-fat-diet-induced obese mice, QUE suppresses plasma leptin and TNF-α levels and reduces elevated macrophage numbers [48]. QUE also improves NF-κB, NADPH oxidase, and antioxidant enzymes, preventing chronic inflammation [48]. In a clinical study, QUE supplementation significantly decreased TNF-α levels, indicating lowered inflammation; however, the serum CRP remained unchanged [49].

CUR decreases TNF-α and interferon-γ (IFN-γ) levels in obese mice, and GIN inhibits TNF- α and MMP-1 in RAW 264.7 macrophages and attenuates nitric oxide production in obese zebrafish, suppressing inflammation [50,51]. A recent clinical trial shows that CUR administered with dietary phytosterols significantly reduces fasting plasma total cholesterol and low-density cholesterol in individuals with elevated blood cholesterol levels [52]. Another clinical trial shows that daily CUR extract consumption can significantly increase high-density lipoprotein cholesterol and reduce low-density lipoprotein cholesterol in subjects with metabolic syndrome [53].

EGCG reduces oxidative stress and inflammation associated with high-fat diet consumption in rats [54]. In addition, EGCG has been shown in vitro to decrease circulating IL-6, TNF-α, ROS, and superoxide dismutase (SOD) levels [54]. In the same study, EGCG also significantly increased SIRT-1 and decreased NF-κB levels [54]. A randomized, double-blind, placebo-controlled trial of 56 obese, hypertensive subjects who received a daily supplement green tea extract or a placebo for 3 months indicated that among other reported parameters, NF-κB levels and CRP levels were significantly lower and the total antioxidant status was increased in the treatment group [55]. These results indirectly point to EGCG’s ability to reduce inflammation and oxidative stress.

PIP has been shown to mitigate insulin resistance in a diabetic obese mouse model. Supplementation by PIP resulted in decreased white blood cell (WBC) count, lipopolysaccharides, and pro-inflammatory cytokines such as galectin-3 and IL-1β [56]. In addition, the 10-week daily treatment with PIP also resulted in the downregulation of mRNA levels of pro-inflammatory cytokines [56].

#### 2.1.2. Diabetes

Per national diabetes statistics, in the United States, 34.2 million people have diabetes and 88 million adults are pre-diabetic [57]. Type 2 diabetes (T2D) is attributed to high saturated fat intake and unhealthy lifestyles [58]. Insulin resistance accompanies T2D when the functional expansion of the islet β cells fails to compensate for insulin deficiency. In T2D, hypoxia and cell death of adipose tissues may occur along with proinflammatory cytokine expression in activated immune cells [58]. Moreover, the expression of inflammatory cytokines is often associated with insulin resistance [59]. Obesity leads to the activation of stress pathways, including Jun N-terminal kinase (JNK) and NF-κB [60]. Additionally, inflammation in the Langerhans beta islet cells of the pancreas results in decreases in beta cell numbers and their function. Studies have highlighted the activation of the inflammasome and IL-1β signaling in the pancreas, leading to T2D [60]. Another aspect commonly seen with T2D is that obesity leads to fat accumulation in organs such as the liver, pancreas, muscle, pericardium, and perivascular regions. This fat accumulation, in turn, activates resident macrophages and switches them to a pro-inflammatory state. M1-type macrophages further amplify the inflammatory cascade, perpetuating chronic inflammation [60]. A clinical trial emphasized the relationship between cytokine elevation and increased risk of developing T2D [61]. Hotamisligil et al. showed that adipose cells in animal models can produce TNF-α, which plays an important role in insulin resistance [62]. Diabetes is associated with decreased IL-10 production, an anti-inflammatory cytokine that reduces IL-6 and TNF-α levels, in turn decreasing insulin receptor tyrosine kinase activity [63].

RES activates SIRT1, improving insulin sensitivity, reducing glucose production, and improving glucose homeostasis in rats [64]. SIRT1 improves peripheral insulin sensitivity following inhibition of protein tyrosine phosphatase 1B in mice [65]. Elevated SIRT1 expression increases islet mitochondrial activities and reduces ROS levels [66]. ROS reduction by RES is noted in a clinical study of T2D patients [67]. In monkeys, RES supplementation increases SIRT1 expression and decreases NF-κB activity, improving insulin sensitivity in visceral white adipose tissue [68]. RES treatment also alleviates insulin resistance by reducing TNF-α, cytokeratin 18, and fibroblast growth factor 21 in patients [69]. In a clinical trial, RES administration significantly decreased total weight, body mass index, and fat mass and increased total insulin secretion [70]. Another trial showed that patients with diabetes and chronic periodontitis, given RES supplementation for four weeks, had significant improvement in insulin resistance and periodontitis status [71].

QUE supplementation significantly suppresses the diabetes-induced activation of the p65/NF-κB, ERK1/2/MAPK pathway, caspase-3, and caspase-9 [72]. QUE also moderately attenuates TNF-α effects, enhancing insulin uptake of glucose in a dose-dependent manner to activate the AMPK pathway in murine skeletal myoblasts, suppressing the NF-ĸB signaling and NO/iNOS system, improving glucose uptake and insulin sensitivity in muscle cells [73].

CUR treatment prevents autoimmune diabetes by inhibiting macrophage infiltration and decreasing NF-κB activation, thereby reducing proinflammatory cytokine secretion in T1D rat models [74]. Further, CUR significantly decreases necrotic cells, IL-6, and CRP levels, reducing liver inflammation by the induction of insulin sensitivity in rats [59]. In T2D patients, CUR decreased adipocyte-fatty acid-binding protein, IL-6, CRP, and TNF-α levels in 100 participants, improving metabolic parameters [75]. Furthermore, diabetic nephropathy showed improvement with CUR treatments by suppressing TGF-β, IL-8, and TNFα expression in T2D patients [76]. One clinical trial with CUR demonstrated a decrease in lipid peroxidation, suggesting a therapeutic potential to restore oxidative damage in obese human subjects within 6 to 12 weeks [77]. 

PIP is often dosed with CUR to improve the bioavailability of CUR. In a randomized, double-blind placebo-controlled trial in T2D patients between 18 to 65 years of age, CUR and PIP were administered with standard of care and compared to standard of care alone over 3 months [78]. Data shows that inclusion of the CUR+PIP supplementation with standard of care resulted in a significant reduction in serum glucose levels and HbA1C levels compared to standard of care alone [78]. However, the inflammatory marker, CRP, levels were similar in both arms [78]. 

GIN treatment in streptozotocin-induced diabetic rats that were administered 6-gingerol by oral gavage for 8 weeks resulted in significantly lower fasting blood glucose levels, hyperlipidemia, and oxidative stress [79]. In addition, the inflammatory response in the 6-gingerol-treated diabetic mice was significantly lower in terms of TNF-α, IL-6, IL-1β, and CRP levels [79]. Lastly, the study also demonstrated that treatment with 6-gingerol ameliorates renal fibrosis and pathological changes associated with this diabetic rat model [79]. A double-blinded, placebo-controlled clinical trial of 50 subjects with type II diabetes was administered daily GIN supplements for 10 weeks [80]. While inflammatory markers were not assessed in this clinical trial, the outcomes indicated that supplementation with GIN resulted in significantly lower fasting blood glucose levels and lower HbA1C levels [80]. 

Data with green tea extract use and predominantly EGCG in T2D has indicated efficacy in vitro, in vivo, and in clinical trials in overcoming insulin resistance, protecting β-islet cells, activating the insulin-signaling pathway, and decreasing inflammation [81,82]. However, other studies have indicated that treatment with EGCG or green tea extracts has no beneficial effects [82]. The authors have postulated that the cultivation of the tea along with its processing may be the main reason for the inconsistent results seen with green tea extracts [82].

#### 2.1.3. Cardiovascular Diseases

According to the American Heart Association, cardiovascular disease (CVD) is the leading global cause of death, accounting for 18.6 million deaths in 2019 [83]. Inflammation is essential in the development of CVD and begins with oxidative modification in the sub-endothelial matrix, triggering an immune response. Inflammatory cytokines and lipid oxidation promote matrix calcification, driving CVD pathophysiology [84,85]. NF-kB activation in human atherosclerotic tissue upregulates pro-inflammatory mediators (TNF-α, IL-6, and IL-8) in atherosclerotic plaques [86].

The impact of RES on cardiovascular functions due to its antioxidant and anti-inflammatory effects has been well studied in vitro [87]. The development of myocardial ischemia and reperfusion injury requires inflammation mediated by cytokines (TNF-α, IL-6, and IL-8), the extracellular matrix, and chemokines regulated by NF-κB. RES treatment has cardioprotective and anti-inflammatory effects in ischemia and reperfusion in rats [88]. Li et al. demonstrated that chronic RES treatment significantly inhibits NF-κB signaling, suppressing TNFα expression and preventing neutrophil infiltration in myocardial ischemia and reperfusion [89]. RES suppression of TNFα improves myocardial ischemia and cardiovascular health in swine and human cardiac cells [90,91]. Moreover, human coronary arterial endothelial cells showed significant inhibition of TNF-α, iNOS, and IL-1β mRNA with RES pre-treatments [92]. RES causes the overexpression of SIRT1, inhibiting the NF-κB pathway [93], neutrophil accumulation, TNF-α expression, and myocardial apoptosis and attenuating myocardial injury in rats [94] and aortic stenosis in humans [95]. Further, a clinical trial showed that daily RES consumption increases serum adiponectin, an anti- inflammatory cytokine, in patients with coronary artery disease [96]. Despite its low bioavailability, RES inhibits NF-κB and AP-1, downregulating pro-inflammatory activation and inhibiting atherothrombotic signals [96]. Additionally, a human trial with RES supplementation for 1 year in 74 patients showed significant cardiovascular protection through the inhibition of CRP, TNF-α, the plasminogen activator inhibitor, and the proinflammatory IL-6/IL-10 ratio [97]. Daily consumption of RES-containing grape extract could increase serum adiponectin and downregulate 27 genes involved in the inflammatory process in peripheral blood mononuclear cells [96].

Numerous in vitro and in vivo studies have demonstrated QUE’s ability to function as a cardioprotective agent [31,98]. Other studies have indicated that QUE also has immunomodulatory effects that attenuate atherosclerotic inflammation through its activity in the TLR-NF-κB signaling pathway [99]. QUE has been shown to be antiatherosclerotic by multiple mechanisms, including decreasing oxidative stress and inhibiting excessive nitric-oxide-induced endothelial damage and its inhibition of the NF- κB signaling pathway, which lead to a suppression in IL-1β, TNF-α, and IL-10 in serum [100]. In addition, studies in atherosclerotic rats have indicated that QUE can significantly downregulate the mRNA expression of TLRs and cytokine-α, further indicating that QUE can have significant effects on chronic inflammation associated with cardiovascular disease [100]. However, the major limitation associated with the use of QUE is its low oral bioavailability and poor aqueous solubility [31,101].

Viral myocarditis, a form of myocardium inflammation, was attenuated by CUR treatments, significantly suppressing myocardial proinflammatory cytokines (TNF-α, IL-6, and IL-1β) in infected mice [102] and rats [103]. Another study confirmed the ability of CUR to suppress myocardial protein levels of NF-κB, IL-1β, and TNF-α, ameliorating cardiac inflammation in rats [104]. Protective effects of CUR in cardiac injury were confirmed through significant suppression of cardiac inflammation (NF-κB, TNF-α, IL-6, IL-1beta, and COX-2) and oxidative stress after four weeks of treatment in rats [105]. A recent study shows heart tissues in CUR-treated rats have lower levels of oxidative stress, inflammatory cytokines (TNF and IL-4), and damage [106]. In a clinical trial, CUR significantly increased vascular NO and reduced oxidative stress, improving endothelial function [107]. Vascular endothelial function was also improved after CUR supplementation in postmenopausal women compared to a control group [108].

PIP has also shown the ability to reduce blood pressure and inflammation-induced CVD. Diets supplemented with PIP show major reductions in oxidative stress, blood pressure, and inflammation in rats [109]. The same study shows protective properties of PIP in rat cardiac histology, displaying a significant increase in mast cell density and inhibition of inflammatory cell infiltration into the heart and liver [109]. PIP pretreatment demonstrated anti-fibrotic effects [110]. Taqvi et al. confirmed blood-pressure-lowering effects by demonstrating a significant dose-dependent reduction of arterial pressure in rats and rabbits [111]. Another rabbit model showed that PIP lowers oxidative stress and reduces oxidative mitochondrial injury in primary rabbit monocytes [112]. The antioxidant and anti-inflammatory effects of PIP in CVD are well described in vitro and in vivo; however, there is a lack of clinical trials utilizing PIP alone to validate these effects [113].

Numerous in vitro and in vivo studies on tea extracts and some on EGCG have demonstrated that these compounds can reduce oxidative stress by activating the p-53 mitogen-activated protein kinase (MAPK) pathway, a hallmark of cardiovascular disease [114,115,116]. A recent review highlights the activities of EGCG in alleviating inflammation through multiple mechanisms, including suppressing blood angiotensin II-associated CRP, regulating the NOTCH pathways, and downregulating TLR4 [117]. Bogdanski et al. reported that a green tea extract capsule in 56 obese, hypertensive subjects, as part of a double-blind, placebo-controlled clinical trial for 3 months, resulted in a significant drop in systolic and diastolic blood pressure, fasting serum glucose, and inflammatory markers TNFα and CRP [55]. A summary of the clinical trials used in cardiovascular disease is discussed elsewhere and does not directly correlate with the anti-inflammatory effects of EGCG or green teas as the clinical end points were related to blood pressure and vasodilation [117]. Like many of the other phytochemicals discussed, green tea and its components, such as EGCG, have poor oral bioavailability, and, thus, their widespread use has been hampered [118].

Limited studies have been conducted on the use of GIN for cardiovascular health. However, a recent study by Wu et al. in porcine coronary arteries using ginger crude extract resulted in the relaxation of coronary arteries via the suppression of nitric oxide synthase and free radical scavenging abilities [119]. A recent review highlights the role of GIN in inhibiting NF-κB and blocking pro-inflammatory cytokines to prevent the activation of the immune system to alleviate cardiovascular disease [120].

### 2.2. Cancers

According to the National Cancer Institute, in 2020, over 1.8 million new cases of cancer will be diagnosed in the USA alone, and over 600,000 will die from cancer [121]. Inflammation plays a role in many cancers, with about a quarter stemming from long-term infections or inflammation [122]. Key regulators in inflammatory processes in cancer include NF-κB, nuclear factor of activated T-cells, hypoxia-inducible factor 1 (HIF-1), and signal transducer and activator of transcription 3 (STAT3) [123,124]. Treatment with natural products has been shown to attenuate some pro-inflammatory and cancer-proliferative effects of these markers and is discussed in the following sections.

#### 2.2.1. Skin Cancers

Chronic inflammation induced by repeated exposure to topical carcinogens or UVB radiation leads to the initiation and advancement of skin cancers by predominantly activating the NF-κB and STAT3 pathways [125,126,127]. Downstream of the activation of the pro-inflammatory cascade, COX-2 converts arachidonic acid to PGE and is upregulated after UVB exposure [125].

Several studies with ginger on skin cancer have been conducted. In one study by Park et al., mice were dosed with 12-O-tetradecanoylphorbol-13- acetate (TPA) [126]. 6-GIN pretreatment decreased tumors and vascular permeability, indicating reduced inflammation [126]. Interestingly, CUR showed a similar trend, with a permeability reduction in the epidermis [126]. A similar study showed that a topical pretreatment of 6-GIN before TPA application reduced inflammation and TNF-α levels [128]. Wu et al. demonstrated that another ginger compound, 6-shogaol, inhibits tumor promotion and inflammatory markers after TPA application, downregulating iNOS and COX-2 expression and suppressing pathways upstream of NF-κB and AP-1 [129]. 6-GIN and CUR reduced iNOS and COX-2 [129]. These studies indicate that topically applied compounds from ginger may prevent or reduce inflammation associated with skin tumors.

Many studies using EGCG have explored its role in the prevention and treatment of skin cancer inflammation. Mice irradiated with UVB and treated topically with EGCG demonstrated decreased skin tumor development, with the inhibition of NF-ĸB cited as a potential mechanism by which this protective effect occurred [130]. EGCG also suppresses the UVB activation of AP-1, a transcription factor involved in both inflammation and tumor promotion [131]. In humans, one study demonstrated the protective effects of green tea polyphenols by applying them topically to the skin of volunteers before UVB exposure and then examining their skin for erythema and measuring DNA damage by assessing for the presence of cyclobutane pyrimidine dimers [132]. DNA damage was reduced with escalating topical green tea polyphenol doses, indicating that green tea extracts may be useful for reducing the risk of skin cancer development after UVB exposure [132]. As the topical application of green tea has demonstrated beneficial results in the prevention and treatment of skin cancers, there is currently a clinical trial (NCT02029352) that is ongoing, assessing the use of topical green tea for treating superficial skin cancer through the Maastricht University Medical Center for patients with basal cell carcinoma.

#### 2.2.2. Colorectal Cancer

According to the CDC, colorectal cancer occurs when cells in the colon or rectum grow out of control. Emerging data indicate that the prognosis for colorectal cancers is poor when coupled with systemic inflammation [133]. Additionally, in 21–41% of the patients with resectable disease, increased serum CRP levels are present, indicating systemic inflammation in response to the tumor [133]. Colon cancer development is associated with inflammation, and inflammation promotes migration and invasiveness [134]. Colitis-associated cancer, a cancer that is directly attributable to chronic inflammation, presents with high concentrations of pro-inflammatory cytokines that can induce mutations in oncogenes and tumor suppressor genes, which, in turn, lead to further disease progression [135]. In many colon cancers, NF-κB and STAT3 are constitutively activated and mediate the immune response and oncogenesis [135]. 

Effects of CUR in colon cancer have been extensively examined. A study by Marjaneh et al. showed CUR decreased the disease activity index and suppressed chronic inflammation in mice [136]. Another study shows PIP, with and without CUR, inhibited mTORC1 activity, which is associated with inflammation and cancer progression [137]. Individual phytochemicals and the combinations thereof decreased TNF-α and COX-2, indicating CUR and its combination with PIP have anti-inflammatory capabilities in colon cancer. A phase IIa clinical trial with CUR was completed in patients with a risk of developing colorectal neoplasia [138]. Patients taking 4 g of CUR by mouth daily for 30 days had significant reductions in aberrant crypt foci, indicating that CUR can be useful in preventing the development of colon cancer [138].

Ginger supplementation was studied in patients at risk of developing colorectal cancer [139]. Of note, patients who received ginger supplementation had decreased expression of MIB-1 and telomerase reverse transcriptase [139]. In a study of simulated mucosal inflammation in guinea pig gastric mucosa, MIB-1 was increased, indicating upregulation during inflammation [140]. Therefore, GIN could decrease inflammation and the development of colorectal cancer and potentially lower inflammation in colorectal cancer patients.

Rats receiving azoxymethane injections treated with QUE had lower levels of inflammatory markers compared to rats treated with saline, demonstrating the potent anti-inflammatory effects of QUE in the colon [141]. Zhang et al. determined that QUE exhibits anticancer effects by inhibiting the NF-κB pathway in colon cancer cells [142]. Han et al. showed that QUE reduces migration and invasiveness of colon cancer by decreasing NF-κB and TLR-4 expression [134]. Inflammatory markers TNF-α, COX-2, and IL-6 were also reduced [134].

#### 2.2.3. Prostate Cancer

According to the American Cancer Society, prostate cancer was the most prevalent cancer among males after skin cancer [143]. A diagnostic tool for prostate cancer is a prostate-specific antigen (PSA) blood test. Although PSA has low sensitivity, an elevation indicates prostate inflammation [144]. Chronic prostate inflammation may influence carcinogenesis and tumor growth [144]. Chronic inflammation in the prostate leads to disease progression and metastases through angiogenesis and the epithelial–mesenchymal transition that drives the tumor microenvironment [145]. The degree of chronic inflammation in the tumor microenvironment has a significant impact on therapeutic outcomes. The pro-inflammatory mediators implicated in prostate cancer include TNF-α, TGF-β, IL-7, IL-2, and macrophage inflammatory protein-1b [145]. 

CUR has the potential to reduce inflammation associated with prostate cancer. Killian et al. investigated the effects of the chemokines CXCL-1 and CXCL-2 in prostate cancer cells and showed that CUR treatment significantly reduces CXCL-1 and CXCL-2 transcription [146]. CUR also inhibits NF-κB by blocking the phosphorylation of NF-κB inhibitor-α and inhibiting the downstream phosphorylation of P65, leading to decreased CXCL-1 and CXCL-2 expression [146]. One study in humans with prostate cancer combined pomegranate, green tea, broccoli, and turmeric into a capsule and monitored PSA levels [147]. Although PSA increased in both treatment and placebo groups, the treatment group increase was significantly lower than the placebo group, indicating polyphenol-rich food may have some benefit in prostate cancer [147]. Another study showed that men with prostate cancer receiving radiotherapy with CUR supplementation had lower superoxide dismutase activity and higher plasma total antioxidant capacity, indicating that supplementation with CUR leads to higher endogenous antioxidant capacities [148].

## 3. Drug Interactions Associated with Phytochemical Use

Several studies show drug–phytochemical interactions, resulting in significant alterations in therapeutic effects, as discussed below. Conventionally, drug metabolism takes place by two consecutive processes: phase I reactions, principally mediated by cytochrome P450 (CYP) enzymes, and phase II reactions that mediate the conjugation of exogenous, endogenous, or reactive metabolites from phase I reactions [149,150]. Uptake and efflux transporters are specialized pumps that determine plasma and tissue concentrations of substances through their involvement in absorption, distribution, and elimination. Efflux is mostly mediated by the ATP-binding cassette (ABC) family of proteins, of which p-glycoprotein (P-gp) is the most widely studied. Though P-gp protects the body from potentially toxic compounds, it limits oral bioavailability, reduces distribution, and enhances the clearance of substances. P-gp inhibition or induction by co-administered drugs can alter the pharmacokinetics and pharmacodynamics of P-gp substrates. Numerous substances are P-gp substrates, inducers, and inhibitors. Modulating drug-metabolizing enzymes, especially CYP and transporters, is recognized as the common mechanism by which phytochemicals affect the pharmacokinetics and pharmacodynamics of drugs. As many patients with chronic inflammatory diseases use phytochemicals, healthcare professionals must be aware of phytochemical–drug interactions. Table 1 summarizes the pharmacokinetic profiles of the major phytochemicals discussed in this article. These data can guide rational dosing in future studies and the evaluation of the therapeutic potential of these compounds.

### 3.1. RES 

A clinical study in 42 healthy volunteers revealed that RES inhibits CYP3A4, CYP2D6, and CYP2C9 activity [151]. Several animal and in vitro studies have shown that RES is a mechanism-based inhibitor of CYP3A4 and a noncompetitive reversible inhibitor for CYP2E1 in microsomes from rat liver and human liver cells containing cDNA-expressed CYPs [152,153]. As CYP3A4 is the most abundant isoform and is responsible for metabolizing approximately 50% of marketed drugs, inhibition by RES could result in major drug interactions due to an increase in oral bioavailability and the accumulation of drugs that are metabolized by this isoform [154]. Inhibition of P-gp transporters by RES is documented in several animal studies. For example, pretreatment of rats with RES significantly increased maximum plasma concentration (C_max_) and the area under the concentration–time curve (AUC), with no significant change in half-life (t_1/2_) and time at which the compound reaches the maximum plasma concentration (T_max_) of fexofenadine, a common antihistamine [155]. The observed increase in the bioavailability of fexofenadine is attributed to the inhibition of P-gp-mediated drug efflux during the absorption phase in the intestine. RES exhibited similar inhibitory effects on the pharmacokinetics of P-gp substrate drugs such as nicardipine and diltiazem [156,157].

### 3.2. QUE

In in vitro studies utilizing fluorescence-based high throughput assays and human c-DNA baculovirus-expressed CYP enzymes, Elbarbary et al. demonstrated a moderate to strong inhibitory effect of QUE on CYP3A4, CYP2C9, and CYP2D6 activity [158]. Consistent with these findings, oral administration of QUE-rich Ginko leaf tablets to rats resulted in the suppression of CYP3A activity [159]. Hyperoside, QUE-3-O-galactoside, strongly inhibits CYP2D6-catalyzed dextromethorphan O-demethylation in human liver microsomes [160]. Treatment of healthy humans with QUE at 1 g daily for 10 days significantly enhanced the exposure of chlorzoxazone, a CYP2E1 substrate, and diclofenac, a CYP2C9 substrate [161,162]. Caution should be taken when QUE is used in combination with therapeutic drugs metabolized by CYP3A4, CYP2C6, and CYP2D6, and dose adjustments may be necessary. Of note, CYP2D6 is the primary isoform that catalyzes the biotransformation of antidepressants and antipsychotics, and extreme caution should be exercised when QUE is co-administered with these agents.

### 3.3. GIN

CYP inhibition assay kits showed 6-, 8-, and 10-GIN potently inhibit CYP2C9 activity, moderately inhibit CYP2C19 and CYP3A4 activity, and weakly inhibit CYP2D6 activity, with 8-GIN being the most potent inhibitor [163]. Although most in vitro studies indicate similar modulation of animal and human CYP enzymes by gingerols, the available data indicate complex interactions when fed as a whole extract versus single agents. For example, Mukkavilli et al. demonstrated that ginger extract (GE) and its active constituents cause the inhibition of CYP isozymes in human liver microsomes in the order of CYP2B6 > CYP2C19 > CYP2C9 > CYP2C8 > CYP3A4 > CYP1A2 [164]. Comparison of IC_50_ values from this CYP inhibition study to the C_max_ of individual gingerols from a human pharmacokinetics study by Zick et al. reveals the C_max_ of the individual agents is at least 3–4-fold lower than their respective IC_50_ values [165]. This may indicate that physiological concentrations of these gingerols are incapable of modulating the activity of CYP enzymes, and risk of clinically relevant drug interactions is low with dietary doses.

### 3.4. CUR

A plethora of data is available to demonstrate the potential inhibitory effect of CUR in several phase I enzymes, especially CYP2C19, CYP2B6, and CYP3A4, and phase II enzymes, especially sulfotransferase (SULT) and UDP-glucuronosyltransferase (UGT) enzymes, both in vitro and in vivo [166]. For example, glucuronidation of acetaminophen, bilirubin, and mycophenolic acid was potently inhibited by curcuminoid extracts in human colon cancer cells [166,167]. Similarly, CUR demonstrated significant inhibition of the sulfation of acetaminophen and 4-nitrophenol in human liver cytosol [166,168]. The inhibitory effect of CUR on the activity of SULT is potent to the extent that the reported IC_50_ of CUR for SULT1A1 is one order of magnitude lower than the peak serum concentration of CUR after therapeutic doses of 4 g to humans [168]. Potent inhibition of intestinal CYP3A4 and P-gp transporters was demonstrated in rats following oral administration of 60 mg/kg CUR for 4 days, as indicated by higher AUC and C_max_ values for oral celiprolol (a P-gp substrate) and midazolam (a CYP3A substrate) [169].

### 3.5. PIP

PIP modulates the activity of several CYP- and phase-II-mediated pathways for drug metabolism. Several studies in healthy human subjects show the effect of PIP in enhancing the bioavailability of orally administered medications, including rifampin, theophylline, and propranolol [170]. Similarly, a single administration of 1 g of black pepper, rich in PIP, resulted in a >2-fold increase in AUC and the elimination t_1/2_ of phenytoin [171]. The PIP-induced increase in oral bioavailability is normally demonstrated as a significant increase in AUC and C_max_ and sometimes a prolongation in elimination t_1/2_ (such as in theophylline). Such alterations could be explained on the basis of the reversible inhibition of drug-metabolizing enzyme and protein transporters, as depicted by Atal et al. [172]. Using the human colon carcinoma cell line (Caco-2) and human liver microsomes, PIP inhibited the transport of digoxin and cyclosporine A, P-gp substrates and was also an inhibitor of human CYP3A-mediated metabolism of verapamil [173]. Modulation of phase II drug-metabolizing enzyme activity is demonstrated in several in vitro and animal studies. Oral administration of 20 μM PIP was found to inhibit the glucuronidation of EGCG in mice, resulting in increased EGCG bioavailability [174]. Similarly, oral supplementation of 50 mg/kg for 16 weeks enhanced the activities of detoxifying enzymes such as glutathione transferase (GST), quinone reductase (QR), and UDP-glucuronosyl transferase (UDP-GT) in mice [175].

### 3.6. EGCG

Previous in vitro animal work and human studies have suggested that green tea extract and catechins, including EGCG, have limited effects on CYP-mediated drug metabolism but might inhibit a wide variety of drug transporters [176]. Studies in human cancer cells suggest that EGCG modulates P-gp function and reverses P-gp-mediated multidrug resistance [177]. Knop et al. demonstrated that green tea and EGCG inhibit the in vitro transport of prototypical substrates of all seven drug transporters investigated (OCT1, OCT2, MATE1, MATE2-K, OATP1B1, OATP1B3, and P-gp), with the highest in vitro inhibitory potency for OATP1B3 [178]. Intravenous EGCG at 5 mg/kg in rats resulted in increased AUC by 2.21-fold, decreased clearance of simvastatin by 2.29-fold, and the inhibition of the OATP1B1 and OAT1B3 [179].

**Table 1:** Pharmacokinetics of select phytochemicals.

Phytochemical	Model	Route and Dose	F	T_max_	C_max_	t_1/2_	Vd	CL	References
curcumin	human	4000 mg/day		1.7	0.51 µM				[180]
		6000 mg/day		2.0	0.64 µM				
		8000 mg/day		1.8	1.78 µM				
		10 g single oral dose		3.3	2.3 ± 0.26 µg/mL	6.8 h			[181]
		12 g single oral dose		3.3	1.73 ± 0.19 µg/mL	6.8 h			
	diabetic rats	PO: 500 mg/kg	47%	15 min	0.06 µg/mL	32.7 min	Vd: 37.5 L/kg	0.85 L/kg	[182]
		IV: 10 mg/kg	100%	5 min	3.15 µg/mL	8.64 min	Vd: 10.6 L/kg	0.83 L/kg	
resveratrol	human	0.4 g single dose trans-resveratrol (fed)	<1%	2 h	42.2 ng/mL	5.6 h		3813 L/h	[183,184]
		0.4 g single dose trans-resveratrol (fasting)		0.5 h	47.3 ng/mL	5.9 h		4249 L/h	
		0.5 g single dose		0.83 h	72.6 ng/mL	2.8 h	9198 L	CL/F: 2235 L/h	[183,185]
								CLr/F: 1.177 L/h	
		1 g single dose		0.76 h	117.0 ng/mL	8.87 h	19,298 L	CL/F: 2593 L/h	
								CLr/F: 0.696 L/h	
		2.5 g single dose		1.4 h	268.0 ng/mL	4.2 h	22,226 L	CL/F: 3471 L/h	
								CLr/F: 0.656 L/h	
		5 g single dose		1.5 h	538.8 ng/mL	8.5 h	66,991 L	CL/F: 66,991 L/h	
								CLr/F: 1.443 L/h	
piperine	rat	170 mg/kg				18.2 h			[186]
		100 mg		T_max1_ 6.3 h T_max2_ 26.0 h	C_max1_ 3.6 µg/mL C_max2_ 1.8 µg/mL	12.85 h			[187]
		PO: 20 mg/kg	24%	2 h	0.983 µg/mL	1.2 h	V_ss_/F: 4.7 L/kg	CL/F: 2.65 L/h/kg	[188]
		IV: 10 mg/kg				8.0 h	V_ss_: 7.0 L/kg	CL: 0.64 L/h/kg	
quercetin	human	PO: 100 mg (in the form of an onion supplement)		0.68 ± 0.22 h	2.3 µg/mL	11.0 h	V_ss_: 128 L	13.3 L/h	[189]
	beagle dog	PO: 10 mg/kg	4%	T_max1_:1.2 h T_max1_: 3.9 h	C_max1_: 0.23 µmol/L C_max2_: 0.23 µmol/L				[190]
6-gingerol	human	PO: 1000 mg		55 min	0.4 µg/mL				[165]
		PO: 1500 mg		60 min	1.69 µg/mL				
		PO: 2000 mg		66 min	0.85 µg/mL	110 min			
		IV: 1.5 mg/kg				1.6 h	V_ss_: 3 L/kg	3.6 L/h/kg	[191]
		IV: 3 mg/kg				1.9 h	V_ss_: 2.8 L/kg	3.6 1.4 L/h/kg	
		IV: 6 mg//kg				1.7 ± 0.8 h	V_ss_: 1.9 L/kg	2.9 0.4 L/h/kg	
EGCG	mouse	IV: 21.8 μmol/kg			unconjugated: 13.6 μmol/L	unconjugated: 237.5 min	unconjugated: 1.6 L/kg	unconjugated: 0.57 L/(min·kg)	[174]
					total: 2.7 μmol/L	total: 211.5 min	total: 8.0 L/kg	total: 0.62 ± 0.17 L/(min·kg)	
		Intragastric: 163.8 μmol/kg	unconjugated: 12.4%	unconjugated: 158 min	unconjugated: 0.04 μmol/L		unconjugated: 465.0 L/kg	unconjugated: 0.45 L/(min·kg)	
			total: 26.5%	total: 90 min	total: 0.28 μmol/L		total: 152.9 L/kg	total: 0.62 L/(min·kg)	
	human	PO: 200 mg		127.1 min	73.7 ng/mL	118.0 min	2009 L	CL/F: 11.4 L/min	[192]
		PO: 400 mg		108.7 min	111.8 ng/mL	162.3 min	4774 L	CL/F: 18.0 L/min	
		PO: 600 mg		180.0 min	169.1 ng/mL	183.7 min	4368 L	CL/F: 12.8 L/min	
		PO: 800 mg		240.6 min	438.5 ng/mL	114.0 min	1044 L	CL/F: 6.0 L/min	

Abbreviations—PO: by way of mouth, IV: intravenously, F: bioavailability, Tmax: time at which the compound reaches maximum concentration in the plasma, Cmax: the maximum concentration in the plasma, t1/2: half-life, Vd: volume of distribution, CL: clearance, Clr: apparent renal clearance, CL/F: apparent total clearance, Vss: volume of distribution at steady state.

## 4. Regulations Associated with Natural Product Use 

### 4.1. Market Availability of Natural Products

Though natural product research has significantly impacted the discovery of drugs available today, there is a sociological rift between modern medicine and natural products. Natural products remain an active source for focused combinatorial approaches to identify new drugs [193]. However, the use of natural products either as extracts or pure phytochemicals remains the purview of the natural products and supplements industry. In the USA, these natural product supplements are regulated under the Dietary Supplement Health and Education Act of 1994 and the Code of Federal Regulations, Title 21, Part 111 [194]. While supplements are federally and locally regulated, structure or function claims are not reviewed by the United States Food and Drug Administration (FDA) [195]. Based on the regulations outlined, dietary supplements include minerals, vitamins, herbs, botanicals, amino acids, concentrates, metabolites, constituents, and extracts or any combination of these components. Per the regulations, these supplements are considered food and not drugs and, thus, are not subject to the rigorous scrutiny that new chemical entities undergo to become drugs. Thus, every new claim in primary literature related to a natural product ingredient or extract spawns a variety of supplements that are marketed to a population at large. 

### 4.2. Regulatory Issues with Currently Approved Natural Products

Though studies have displayed the benefits and relative safety of natural products, there are issues regarding their safety due to poor regulation, lack of education, and widespread availability. Consumers that take these products may not understand that the dosage forms and doses chosen may not produce the intended effect. In fact, where polypharmacy is present, the inclusion of these supplements may result in adverse drug events [196]. Thus, it is critical that health care practitioners be aware of the limitations of the approval process for dietary supplements. While there are stringent requirements for drug approval, regulations for dietary supplements are more relaxed. Manufacturers are responsible for substantiating the safety and claims of natural products, which presents a conflict of interest, and they are not required to ensure each batch and/or lot is similar in potency. This can cause problems with safety, as consumers may take variable amounts with each batch, and could contribute to drug–drug interactions. Manufacturers are not responsible for providing the FDA information on how substances are quantified, nor are they required to substantiate safety. It is not necessary to register supplements with the FDA, and there are usually no identifying markers on tablets or capsules. This weak regulatory environment may lead to poor manufacturing practices, compromising patient safety. In modern disease therapies, natural products are often used indirectly. For example, 48.6% of cancer therapies developed from the 1940s to date are derived from natural products [197]. Natural products used in the pharmaceutical field are regulated and evaluated by the FDA Center for Drug Evaluation and Research. Natural products used as therapies for diseases must undergo the same strict requirements as synthetically produced drugs.

## 5. Natural Product Informational Databases

Growing popularity in phytochemical studies necessitates a systematic method of recording information about natural products and their derivatives. Extensive research has provided a wealth of knowledge that scientific entities have compiled into databases: SuperNatural, Dictionary of Natural Products, and Natural Medicines Comprehensive Database, among others. In the practice setting, these databases are a resource for healthcare providers to evaluate indications and efficacy from clinical trials so they can make appropriate recommendations. As the FDA labels natural health products as dietary supplements, there is a laxer set of regulations, resulting in multiple manufacturers producing the same compound in varying purities, doses, and/or concentrations, presenting challenges for consumers [198]. Most insurance companies prohibit providers from making suggestions on natural non-drug therapy, suggesting natural medicine is not accepted as a replacement for mainstream medicine.

## 6. Discussion and Future Directions

While the highlighted studies elucidate mechanisms useful in mitigating these disease states with hallmarks of chronic inflammation, their clinical presence is lacking. There are major gaps in our understanding that need to be addressed before the use of these supplements can be made the standard of care in conditions such as metabolic syndrome or cancer. These include a paucity of clinical trials to establish dose; the lack of standardized design for in vivo and clinical trials to effectively compare doses with the responses observed; the low solubility and poor oral bioavailability of many of these compounds (Table 2), raising questions regarding their use in the short term, especially with the prevalent data indicating that chronic daily use may be needed to see effects; and, lastly, the potential interactions that may occur when these phytochemicals are used with conventional therapeutics in these concomitant disease states. 

Table 2 summarizes the physicochemical properties of the selected phytochemicals foundational to formulation and oral bioavailability. These properties include molecular weight, partition coefficient (log P), and intrinsic aqueous solubility. Further, these properties represent formulation challenges that are only partially addressed in clinical studies. For example, as of July 2018, there were 179 clinical trials on CUR. Of these, only 14 focused on improving CUR bioavailability. The disconnect between formulation and therapy is multifactorial: (1) an individual phytochemical may not account for all plant health benefits, (2) formulation may not be optimized, and (3) human variability exists in the metabolism and expression of inflammatory biomarkers. 

Combinatorial approaches to optimize the delivery of phytochemicals have had recent successes. Shaikh et al. have developed a nanoparticle formation of curcumin and piperine that improved oral bioavailability 9-fold when compared to that of curcumin alone, establishing piperine as an absorption enhancer [206]. Despite significant advances in overcoming challenges in translation from preclinical studies to safe and effective use of drugs, drug development attrition (80–90% according to the National Institutes of Health) remains a clear and present danger to public health and the national economy [207]. Technologies such as the use of absorption enhancers and other drug delivery platforms (e.g., nanotechnology, biomedical devices, controlled delivery with respect to time and space) represent opportunities for the optimal and accessible delivery of therapeutics [208]. Continued science-based studies, integrating physiochemical and formulation expertise, are required to ensure safety and efficacy for natural product supplements.

## 7. Conclusions

Inflammation plays a protective role against infection and promotes regeneration; however, balanced inflammation activity, critical for homeostasis, is hard to achieve, especially in aging adults and comorbidities. Accumulation of immune cells and overproduction of inflammatory cytokines are responsible for the pathogenesis of numerous chronic diseases. Interest in phytochemicals is rising due to their antioxidant and anti-inflammatory properties in defending against the inflammation associated with these diseases. Health benefits from phytochemicals such as RES, QUE, CUR, PIP, EGCG, and GIN show that these agents can be developed for treating chronic disease-related inflammation. Despite numerous studies in vitro and in vivo, the number of clinical trials for phytochemicals in inflammation related to chronic diseases is limited. Considerations for the potential use of phytochemicals as therapeutic agents include bioavailability and solubility limitations. More refined, translational molecular approaches are required to realize the full potential of phytochemicals for the treatment of these disease states.

## Figures and Tables

**Table 2:** Physicochemical properties and oral bioavailability for selected phytochemicals.

Phytochemical	MW	Log P	Intrinsic Aqueous Solubility	Oral Bioavailability
RES [199]	228.24	3.1	100 µg/mL	<<1%
QUE [200]	302.23	1.5	60 µg/mL	4%
CUR [182,201]	368.40	3.2	Insoluble in water	0.47%
PIP [188,202]	285.34	3.5	40 µg/mL	24%
GIN [165,203]	294.4	2.5	80 µg/mL	low
EGCG [204,205]	458.4	1.2	32.8 µg/mL	0.1%
